# Investigating the Use of Digital Health Technology to Monitor COVID-19 and Its Effects: Protocol for an Observational Study (Covid Collab Study)

**DOI:** 10.2196/32587

**Published:** 2021-12-08

**Authors:** Callum Stewart, Yatharth Ranjan, Pauline Conde, Zulqarnain Rashid, Heet Sankesara, Xi Bai, Richard J B Dobson, Amos A Folarin

**Affiliations:** 1 Department of Biostatistics and Health Informatics Institute of Psychiatry, Psychology and Neuroscience King’s College London London United Kingdom; 2 Institute of Health Informatics University College London London United Kingdom; 3 Maudsley Biomedical Research Centre South London and Maudsley NHS Trust and King's College London London United Kingdom; 4 Health Data Research UK London University College London London United Kingdom; 5 NIHR Biomedical Research Centre University College London Hospitals NHS Foundation Trust London United Kingdom

**Keywords:** mobile health, COVID-19, digital health, smartphone, wearable devices, mental health, wearable, data, crowdsourced, monitoring, surveillance, observational, feasibility, infectious disease, recovery, mobile phone

## Abstract

**Background:**

The ubiquity of mobile phones and increasing use of wearable fitness trackers offer a wide-ranging window into people’s health and well-being. There are clear advantages in using remote monitoring technologies to gain an insight into health, particularly under the shadow of the COVID-19 pandemic.

**Objective:**

Covid Collab is a crowdsourced study that was set up to investigate the feasibility of identifying, monitoring, and understanding the stratification of SARS-CoV-2 infection and recovery through remote monitoring technologies. Additionally, we will assess the impacts of the COVID-19 pandemic and associated social measures on people’s behavior, physical health, and mental well-being.

**Methods:**

Participants will remotely enroll in the study through the Mass Science app to donate historic and prospective mobile phone data, fitness tracking wearable data, and regular COVID-19–related and mental health–related survey data. The data collection period will cover a continuous period (ie, both before and after any reported infections), so that comparisons to a participant’s own baseline can be made. We plan to carry out analyses in several areas, which will cover symptomatology; risk factors; the machine learning–based classification of illness; and trajectories of recovery, mental well-being, and activity.

**Results:**

As of June 2021, there are over 17,000 participants—largely from the United Kingdom—and enrollment is ongoing.

**Conclusions:**

This paper introduces a crowdsourced study that will include remotely enrolled participants to record mobile health data throughout the COVID-19 pandemic. The data collected may help researchers investigate a variety of areas, including COVID-19 progression; mental well-being during the pandemic; and the adherence of remote, digitally enrolled participants.

**International Registered Report Identifier (IRRID):**

DERR1-10.2196/32587

## Introduction

### Background

The COVID-19 pandemic has brought about widespread and drastic changes to people’s lives, work, and health resulting from infection by SARS-CoV-2 as well as the public health and social measures (PHSMs) that were introduced to limit the disease. It is important to not only understand how and under what circumstances the disease itself spreads but also understand the holistic impact of the pandemic.

Although many people are resilient to the conditions imposed by the pandemic, previous instances of disease outbreaks [1] and quarantines [2] have been associated with negative psychological outcomes. Postinfection conditions that followed previous coronavirus outbreaks include posttraumatic stress disorder, depression, anxiety, and confusion, among others. Similarly, quarantine has been associated with several conditions, including stress [3], posttraumatic stress disorder [4,5], and depression [4,6]. A longer duration of quarantine is associated with worse psychological outcomes [2]—a potentially pertinent fact given the protracted period of the COVID-19 pandemic. Additionally, the stigma of disease and the hazards that many face may differ among different people in different occupations or sociodemographic groups [7].

More recently, the presence of persistent symptoms following acute COVID-19 illness has received increased attention. Around 20% of people in an Office for National Statistics survey from the United Kingdom who had a positive COVID-19 test result reported symptoms lasting at least 5 weeks, and 10% reported symptoms lasting at least 12 weeks [8]. The symptomatologic groups, which are formed by people with persistent illness following SARS-CoV-2 infection, have not been fully determined. Preliminary studies show a multitude of symptoms with various levels of co-occurrence, including persistent respiratory issues, fatigue, psychological and neurological symptoms, and fever [9-11]. The presence of these long-term symptoms is often referred to as *long COVID*.

Mobile health (mHealth) as a field is well suited to the unique problems that have been encountered during the COVID-19 pandemic [12,13]. The need for social distancing and wide-scale quarantines precludes many studies that require direct physical contact with participants. Apart from the ability to continue where other study and data collection methods have been limited, mHealth technologies also offer various advantages. The pervasive nature of mobile phones and wearable fitness devices allows for a fine-grain, second-by-second level of detail as well as prolonged periods of continuous monitoring, which are useful because although the pandemic has been long in duration, it has often been punctuated by acute events, such as infection or the introduction of public health measures. Moreover, the fine resolution of such data provides a more comprehensive view of a person’s health and behavior. Historic fitness, health, and activity records are often connected to a person’s web-based accounts. Participants are able to donate such data, which can be used to better understand changes related to participants’ prepandemic activities and health, their preinfection status, and the duration required to recover to preinfection baseline. Finally, passive data sets collected in this manner have the benefit of being in a standardized format, regardless of their country or institution of origin, and larger numbers of potential participants can be quickly reached through digital methods compared to those reached through more traditional recruitment strategies.

Various previous and ongoing studies have demonstrated the ability to monitor long-term mental well-being [14,15] and track the prevalence of flu-like disease [16] through the use of remote monitoring technologies (RMTs). Such technologies therefore appear to be a useful lens through which to investigate the COVID-19 pandemic, and multiple initiatives have been set up by several groups [17-19].

### Objectives

To investigate some aspects of the COVID-19 pandemic, we launched the Covid Collab study in April 2020. The study is a crowdsourced initiative [20] that will involve remote enrollment. It will use a cross-platform phone app to deliver surveys; allow for the input of COVID-19–related data; and allow participants to connect to third-party sources of wearable data, such as Fitbit LLC. By prospectively collecting regular mental well-being and COVID-19 survey data alongside historic and ongoing health-related wearable device data, we hope to address the following objectives.

We will determine whether remote monitoring can provide data on COVID-19 states with objective, measurable differences. Wearable device data have previously been used to predict the prevalence of influenza-like illnesses [16] and can therefore potentially be used to better understand levels of infection and persistent postsequelae symptoms. We aim to assess the feasibility of detecting acute infections, wellness, and long COVID symptoms at a personal and population level.

We will also stratify and define patterns of symptoms of COVID-19 and any postacute infection illness. Self-reported symptoms and objective measures of activity from wearables will be used to identify any groups or patterns of symptoms, especially those among the nonhospitalized population, which has been less visible and easy to recruit in many studies.

We also aim to identify risk factors and causes of COVID-19, long COVID, and the severity of illness. The incidence of COVID-19 and the likelihood of a person developing persistent symptoms following infection will be investigated with respect to a person’s state prior to enrollment, which will be based on sociodemographic information; participants’ prior medical histories; and wearable- and phone-derived information, such as activity levels, heart rates, and sleeping patterns.

Finally, we will investigate mental well-being throughout the pandemic. Alongside measures of SARS-CoV-2 infection, we will also collect regular responses to mental well-being surveys. We will describe trajectories of mental well-being in response to illness and PHSMs during the pandemic as well as identify risk and protective factors.

## Methods

### Study Design

The Covid Collab study is a crowdsourced observational study that will involve remote enrollment. Covid Collab aims to collect wearable device data, phone data, and survey responses from a large number of self-enrolled participants. This is an observational population study with several structures available for particular objectives. Cross-sectional comparisons will involve drawing cases and controls from participants who have and have not reported illness during the course of the study. By conducting individual longitudinal comparisons and participant-specific models, baseline measurements will be compared against measurements from different stages of COVID-19 (ie, acute infection and postinfection) or from periods of interest (eg, vaccination periods and lockdowns).

### Recruitment

Recruitment started in April 2020 on a small scale, and large-scale recruitment began in June 2020. Given the crowdsourced nature of the study, participants will be able to enroll from anywhere. However, because of the location of our research group, the majority of the promotional activities that have been carried out have targeted people within the United Kingdom. The study is open to enrollment for any person over the age of 18 years who uses a smartphone and, optionally, a wearable fitness device. Participants without a fitness device will still be able to complete COVID-19 and mental health surveys.

Participants will enroll within the Mass Science app—the study app for Covid Collab. During enrollment, the participants will be provided with in-app study information, an in-app consent form, and a basic demographics survey. Directly following enrollment, the participants will go through an onboarding procedure. First, participants will complete a more in-depth demographic survey for collecting information on age, gender, ethnicity, height, weight, previous and existing medical conditions, employment status and whether there has been a change in employment status during the pandemic, and marital status. Second, participants will receive prompts for optionally turning on the location data sharing function in the background of their smartphones throughout their involvement in the study. They will also receive prompts for connecting their wearable device accounts to facilitate wearable device data collection.

### Platform and Mass Science App

**Figure 1:**
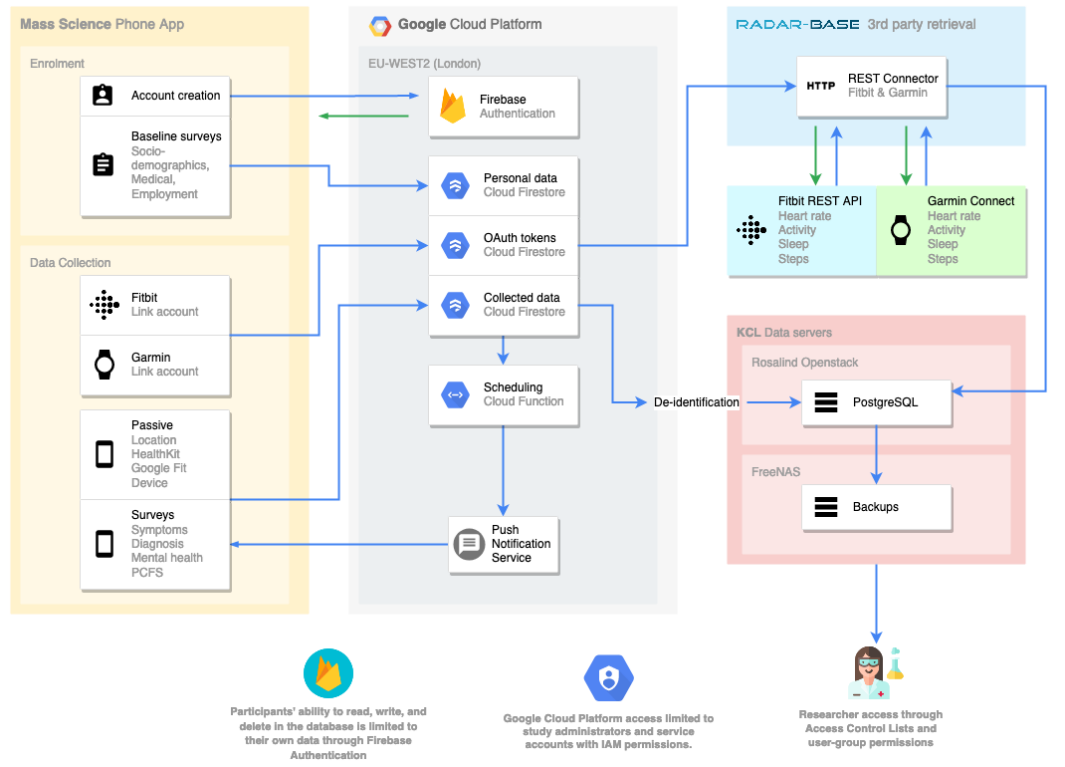
An overview of the data collection platform that will be used in the Covid Collab study. API: application programming interface; IAM: Identity and Access Management; KCL: King’s College London; OAuth: Open Authorization; PCFS: Post–COVID-19 Functional Status; REST: Representational State Transfer.

To facilitate the study, we used pieces of the Remote Assessment of Disease and Relapse (RADAR)–base mHealth data [21] collection platform, alongside services from Google Cloud Platform, as the data collection back end and a custom-built app for remote enrollment and participant interaction (Figure 1).

The Mass Science app is a cross-platform smartphone app that was developed for the Covid Collab study using Flutter. Its key functionalities include providing prospective participants with the ability to enroll in the study; delivering scheduled surveys; allowing participants to input information related to SARS-CoV-2 infection and vaccination; collecting wearable device data either directly from phones or by requesting access to participants’ data through third-party application programming interfaces (APIs); and collecting phone data, including location information. The collection of each data type (eg, location) will be optional. This will allow people to provide data that they are comfortable to share.

Google Cloud Platform [22] comprises the majority of the back end. User authentication will be managed through Firebase Authentication (Google LLC), survey scheduling will be managed through Cloud Functions and Firebase Cloud Messaging (Google LLC), and the initial collection of phone data and surveys will be conducted through Firestore (Google LLC).

RADAR-base is a general mHealth data collection platform that has been used in several RMT studies [14,23,24]. It comprises several modular applications. Some wearable device companies provide access to their customers’ data through an API (a set of definitions and protocols that ease programmatic access to services). We will use the RADAR-base Representational State Transfer Connector to retrieve wearable device data from the Fitbit Web API (Fitbit LLC) and Garmin Health API (Garmin Ltd).

### Procedures and Data Collection

#### Surveys

A number of baseline, on-demand, and scheduled surveys (Table 1) will be included in the study and completed by participants through the Mass Science app. Sociodemographic and medical information will be collected at baseline. Mental health questionnaires—the Patient Health Questionnaire-8 (PHQ-8) scale [25] for symptoms of depression and the General Anxiety Disorder-7 (GAD-7) scale [26] for symptoms of anxiety—will be made available, and participants will be prompted to complete these questionnaires every 2 weeks. A questionnaire on COVID-19 and long COVID symptoms and a visual analog happiness and energy scale will also be made available. These can be completed on demand, but participants will also be prompted biweekly to complete them. COVID-19 diagnosis and vaccination information can be submitted at any time. Following a reported COVID-19 diagnosis, participants will be prompted to fill in the Post–Covid-19 Functional Status scale [27]. Prompts regarding when a scheduled survey is available will be delivered through Firebase Cloud Messaging push notifications, which will appear as notifications on participants’ phones.

**Table 1:** The surveys that will be collected during the study.

Questionnaires		Purpose	Frequency
**Baseline questionnaires**			
	Covid Collab demographics (Multimedia Appendix 1)	To collect demographic data	Baseline
	Covid Collab comorbidities (Multimedia Appendix 1)	To collect data on disorders and comorbidities	Baseline
**Scheduled questionnaires**			
	Post–COVID-19 Functional Status scale [27]	A fast ordinal scale for the evaluation of post–COVID-19 functional status	Fortnightly following diagnosis
	COVID-19 symptoms (Multimedia Appendix 1)	To catalog acute-phase and lingering COVID-19 symptoms and long COVID symptoms	Twice weekly and on demand
	General Anxiety Disorder-7 [26]	To identify probable cases of anxiety and to determine the severity of symptoms	Fortnightly
	Patient Health Questionnaire-8 [25]	To assess the severity and presence of symptoms of depression	Fortnightly
**On-demand questionnaires**			
	Diagnosis (Multimedia Appendix 1)	Self-report diagnosis questionnaire	On demand
	Vaccination (Multimedia Appendix 1)	Vaccination survey	On demand

#### Wearables

Wearable device data will be collected through 2 methods. First, participants can connect their web-based accounts, thereby allowing us to collect data from the wearable vendors’ HTTP API. Both Fitbit LLC and Garmin Ltd will provide data access through this method by allowing users to authorize Covid Collab to access their data through the companies’ respective APIs. In this case, data can be retrieved directly from a server. Second, we can retrieve data via users’ smartphones by using Apple HealthKit (Apple Inc) [28] and Google Fit (Google LLC) [29]. In this case, data will be uploaded to Firestore alongside other phone data.

The exact data types that will be available will depend on the devices that the participants use, what the wearable device manufacturers make available, and what the users choose to authorize when allowing access to their wearable data. Where available, we will collect intraday and summary heart rate, step count, and activity data; sleep data; and other physical and health information, such as height and weight. If a participant does not own a wearable device, they will still be able to provide survey responses and phone data through the Mass Science app.

We expect that some participants will have existing data for the periods of time preceding enrollment and the pandemic. After they connect their wearable device accounts, we will retrospectively collect wearable device data from January 2019, where available. Prospective data will be retrieved as they become available.

#### Location

Geographic position data will be collected from consenting participants’ phones. To reduce battery use, a location point will be recorded only when movement is detected and not when participants are stationary. Raw location data are highly sensitive. As such, they will be stored separately, and only deidentified features and summary statistics will be accessible to researchers. Following a change in stance by the Google Play Store (Google LLC) in January 2021, location collection was discontinued in subsequent updates of the Android app.

#### Data Enrichment

Analyses will require the enrichment of the data through the incorporation of publicly available data sets. Primarily, this will be performed via the contextualization of location data by using the CORINE (Coordination of Information on the Environment) Land Cover data set [30] and OpenStreetMap (OpenStreetMap Foundation) and via the incorporation of public and social measures from the World Health Organization PHSM database [31].

#### Data Management

All data will be stored and encrypted, and personal information will be stored in a separate database. Location data will be deidentified via the aggregation of raw geographic coordinates into features. Access to personal information will be limited strictly to study administrators for administration purposes (eg, to delete data at the request of a participant). Researchers’ access to the anonymized data set will be limited through access control lists. Participants can choose to allow us to share anonymized versions of their data in a larger public data set, which will be made available at a later date.

### Statistical Analysis

#### Data Exclusion and Absence

As a crowdsourced study involving the optional sharing of different modalities of data, we expect that there may be greater data missingness and participant attrition than those in studies that involve more direct patient contact and engagement. Different objectives may require different exclusion criteria. For example, determining wearable biomarkers for COVID-19 may only require a connected device and a single COVID-19 diagnosis survey, while characterizing trajectories of mental well-being would require multiple PHQ-8 and GAD-7 responses from a single participant.

Rates of participation, adherence, and dropout will be examined with respect to sociodemographics, time points during the pandemic, and participants’ concurrent health. Additionally, patterns of user engagement will be characterized to show how participants may interact in similar studies and what drives engagement. User engagement will be determined based on completion rates for the prompted surveys.

#### Characterizing COVID-19 and Long COVID Symptomology

We will describe and define subgroups for symptoms of COVID-19 and long COVID through the clustering of self-reported symptoms. This will include a time-independent view of all symptoms throughout the illness as well as time-dependent clustering to investigate how the disease progresses. A latent class analysis will be used to group time-independent symptoms. A cluster analysis will be conducted on symptom severity data (4-point Likert scale). The optimal number of latent classes will be selected based on the Bayesian information criterion. Time-dependent symptom clustering will be carried out by using mixture latent Markov models. The classes will be described with respect to the frequency of symptoms and their prevalence in different sociodemographic groups.

#### Risk Factors for Severe COVID-19 and Long COVID

Risk factors will be assessed by conducting a logistic regression between participants with long COVID symptoms and participants who had COVID-19 but did not experience persistent symptoms. A logistic regression between participants with COVID-19 who self-report severe symptoms (based on a 4-point Likert scale) and those who self-report mild symptoms or are asymptomatic will also be conducted. Predictors will include sociodemographics, smoking status, medical history, and measures of health and behavior derived from the RMT passive data streams (eg, historic and contemporary activity levels and heart rates).

#### Disease State Classification

By using the identified clusters of symptoms, we will explore RMT parameters that can be used to classify COVID-19. This analysis will involve using conventional machine learning methods, including support vector machines and random forests, in combination with feature selection and fusion approaches, as well as more contemporary deep learning methods.

#### Trajectories and Classification of Mental Well-being

The primary mental health outcome measures will be the PHQ-8 and GAD-7 for depression and anxiety, respectively, which participants will be prompted to complete every 2 weeks. Additionally, a visual analogue scale for happiness and for energy will be included alongside the biweekly symptoms questionnaire.

Mental well-being will be investigated from several viewpoints. First, we will analyze how mood changes in response to and following a SARS-CoV-2 infection. Second, we will determine how mental well-being has been affected throughout the pandemic for the entire cohort in relation to public health measures and by taking into account levels of activity and information on location (eg, time spent outside, home stay duration, or local population density). Finally, we will assess the feasibility of using machine learning approaches to predict low mood on the basis of passive wearable and phone data.

## Results

The Covid Collab study began in April 2020, and large-scale recruitment began in earnest in June 2020. As of June 2021, there are over 17,000 participants. Of those, 11,350 have a connected wearable device, and 16,350 have completed at least 1 survey. An interim analysis is expected to be complete by July 2021. The publication of the final analyses is expected to occur by December 2022 but may depend on the evolution of the COVID-19 pandemic.

## Discussion

Remote monitoring is a promising avenue for understanding COVID-19 and the effects of the pandemic. Our study has multiple advantages, including the availability of historic wearable device data, the ability to reach a wide range and large number of people, and the high resolution of data. However, there are also a number of limitations to the study.

Although crowdsourced recruitment is technically open to all, it is likely that there will be bias. The study is only reachable by those who own a smartphone, and a person who already owns a wearable device may be more likely to take part in the study. Both of these populations may be skewed, in some respect, relative to the general population. Moreover, different segments of the population may be more likely to seek out and engage with scientific studies of this kind. For example, within our currently enrolled cohort, 68.6% (11,840/17,255) of participants are female. It will be important to quantify the composition of the cohort and determine how the composition relates to the known COVID-19 incidence rates among different groups, study adherence rates, and data completion rates within the study.

Participant attrition is present in many internet-based studies [32]. As previously mentioned, due to the nature of a study involving remote enrollment and little to no personal interaction, we may expect higher attrition rates than those in studies with different enrollment strategies or methods for promoting participant interaction and engagement [33]. A “history view” screen was implemented in the app. It shows previous mood responses to allow for the direct return of results to participants. However, other studies have used other methods for promoting participant engagement that are not present in our study largely due to development time limitations. Such methods include different notification strategies [34,35] and gamification [36,37].

Another limitation is imposed by the evolving nature of the pandemic and our knowledge of COVID-19; in response to new information, we may be required to change aspects of or add to the protocol. For example, long COVID symptoms and the Post–COVID-19 Functional Status scale were added recently, as more evidence of persistent impairment following SARS-CoV-2 infection has emerged. Time constraints also require us to balance the introduction of features with the need to recruit participants at an earlier stage. For example, the use of the Garmin Health API was recently included in the protocol. This may have limited the prior recruitment of users of Garmin devices. However, current and prospective participants with Garmin devices will still be able to donate historic data connected to their accounts.

There are several similar ongoing studies throughout the world. Although our participants may overlap with those of other studies, each study is fairly well geographically separated. Although we are recruiting participants from throughout the world, as a UK-based group, our outreach and ability to connect with potential participants are greatest in the United Kingdom. Given the similarity of the collected data and the loose alignment of questionnaires, there is potential for collaboration or meta-analysis.

Overall, the introduced study ought to provide an angle through which to view the mental and physical health of a population throughout the COVID-19 pandemic. Using historic and ongoing wearable and mHealth data should allow for more thorough precision health models to be built and enable us to understand how prior lifestyles have affected the risk of developing COVID-19, long COVID symptoms, and mental health issues.

## Appendix

Multimedia Appendix 1 Definitions for the unpublished questionnaires that will be used in the Covid Collab study.

## Abbreviations

API: application programming interface
CORINE: Coordination of Information on the Environment
GAD-7: General Anxiety Disorder-7
mHealth: mobile health
NHS: National Health Service
NIHR: National Institute for Health Research
PHQ-8: Patient Health Questionnaire-8
PHSM: public health and social measure
RADAR: Remote Assessment of Disease and Relapse
RMT: remote monitoring technology
