# Supplementary material for: Investigating the Use of Digital Health Technology to Monitor COVID-19 and Its Effects: Protocol for an Observational Study (Covid Collab Study)
Source: JMIR Res Protoc. 2021 Dec 8;10(12):e32587. doi: 10.2196/32587 (PMC8658240; doi:10.2196/32587)
Supplement: Multimedia Appendix 1 [file resprot_v10i12e32587_app1.docx]

# S1. Questionnaires

## Symptoms

| **Task name** | **Task type** | **Task text** | **Choices** | **Additional information** |
| --- | --- | --- | --- | --- |
| Mood | Slider(s) | How are you feeling today? | Happiness  Energy | Rate arousal (energy) and valence (happiness) between -1 and 1 |
| COVID symptoms | List of symptom ratings | Please rate the severity of any Covid-related symptoms you are currently experiencing. | ‘Fever’,  ‘Cough’,  ‘Difficulties breathing’,  ‘Anosmia’,  [Free text entry] | Each symptom is rated between 0-3 (None, Mild, Moderate, Severe) |
| L-COVID symptoms | List of symptom ratings | Please rate the severity of any Long-Covid related symptoms you are currently experiencing, even if you are not known to previously have had a Covid-19 infection | ‘Fatigue’,  ‘Difficulty thinking’,  ‘Difficulty sleeping’,  [Free text entry] | Each symptom is rated between 0-3 (None, Mild, Moderate, Severe) |

## Diagnosis

| **Task name** | **Task type** | **Task text** | **Choices** | **Additional information** |
| --- | --- | --- | --- | --- |
| Recipient | Radio button | Who received a diagnosis?  Did you or another person in your household receive a Covid-19 diagnosis? | ‘I have been diagnosed’,  ‘A person I live with has been’ diagnosed |  |
| Diagnosis type | Radio button | Type of diagnosis  What type of diagnosis was performed? | ‘PCR - Tests for a current infection’  ‘Antibody - Tests for a previous infection’,  ‘Self-diagnosed or assumed based on symptoms’,  ‘Lateral flow test - Rapid antigen test for a current infection’ |  |
| Diagnosis date | Datetime | Date of Covid-19 diagnosis.  On what date did you receive the diagnosis? | Date field |  |
| Illness date | Datetime | Date of Covid-19 illness  On what date were you first ill or did you first notice symptoms? | Date field |  |

## Vaccination

| **Task name** | **Task type** | **Task text** | **Choices** | **Additional information** |
| --- | --- | --- | --- | --- |
| Vaccine type | Dropdown choice | Vaccine type  Please select the vaccine you received from the list below | ‘Pfizer-BioNTech’,  ‘Moderna’,  ‘Oxford-AstraZeneca’,  ‘Don’t Know’,  ‘Other’ |  |
| Vaccine date | Datetime | Vaccination date  On what date did you receive this vaccine? | Date field |  |
| Vaccine dose | Radio button | Vaccine dose  Did you receive an initial dose or a booster shot? | ‘Initial dose’,  ‘Booster shot’ |  |

##

## Social demographics and medical history

| **Task name** | **Task type** | **Task text** | **Choices** | **Additional information** |
| --- | --- | --- | --- | --- |
| ethnicity | Multichoice | Please select any of the below ethnicities which you identify with.  You can select multiple choices or type in an ethnicity that you identify with but is not listed. | 'Prefer not to say',  'Arab',  'Black',  'Central Asian',  'East Asian',  'South Asian',  'South-east Asian',  'White',  [Free text entry] |  |
| employment | Multichoice (tickbox) | Employment  What was your employment status prior to the pandemic? (tick all that apply) |  |  |
| employment change | Multichoice (tickbox) | Changes to employment  Has your employment status changed since the pandemic began? (tick all that apply) |  |  |
| marital status | Dropdown | Marital status  What is your current marital status? |  |  |
| children | Dropdown | Children  Do you have children? | ‘Yes’,  ‘No’ |  |
| living situation | Multichoice (tickbox) | Living situation  What is your current living situation?  Please check any that apply to you. | ‘Alone’,  ‘With family’,  ‘Partner or spouse’,  ‘Family of partner or spouse’,  ‘Housemates’,  ‘With children under 18’,  ‘With children over 18’,  ‘Different situation than normal’,  ‘Different country than normal’,  ‘Other’,  ‘Prefer not to say’ |  |
| height weight | text input | Height and weight  What is your height (in centimetres) and weight (in kilograms) | N/A |  |
| physical health history | Multichoice | Have you ever been diagnosed with one or more of the following?  You can select multiple options or type in additional problems that are not listed. | 'Asomnia',  'Asthma',  'Cancer',  'Cerebrovascular disease',  'Chronic kidney disease',  'Cystic fibrosis',  'COPD (chronic obstructive pulmonary disease)',  'Diabetes (Type 1)',  'Diabetes (Type 2)',  'Heart conditions (Coronary artery disease, cardiomyopathy, etc)',  'Hypertension or high blood pressure',  'Immunocompromised state',  'Liver disease',  'Neurological condition (e.g. dementia)',  'Obesity',  'Pulmonary fibrosis (scarred lung tissues)',  'Sickle cell disease',  'Thalassemia', |  |
| mental health history | Multichoice | Have you ever been diagnosed with one or more of the following mental, behavioural, neurodevelopmental or sleep-wake conditions?  You can select multiple options or type in additional problems that are not listed. | 'Prefer not to say',  'Don't know',  'Autism',  'Dementia',  'Attention deficit/hyperactivity disorder (ADHD/ADD)',  'Bipolar, mania, hypomania, or manic-depression',  'Depression',  'Seasonal affective disorder (SAD)',  'Anxiety, nerves, or generalised anxiety disorder',  'Social anxiety or social phobia',  'Agoraphobia',  'Specific phobia (e.g. of flying)',  'Obsessive-compulsive disorder (OCD)',  'Body dysmorphic disorder (BDD)',  'Other obsessive-compulsive related disorder',  'Dissociative disorder',  'Somatic symptom disorder',  'Anorexia nervosa',  'Bulimia nervosa',  'Other feeding or eating disorder',  'Narcolepsy',  'Insomnia disorder',  'Hypersomnolence disorder',  'Sleep apnea',  'Parasomnia (e.g. sleepwalking, sleep terrors)',  'Conduct disorder',  'Substance related disorder',  'Personality disorder',  'Schizophrenia',  'Schizoaffective disorder',  'Any other type of psychosis or psychotic illness',  'Panic disorder',  'Panic attacks',  'Post-traumatic stress disorder (PTSD)' |  |
